# Effects of Thymoquinone on Small-Molecule Metabolites in a Rat Model of Cerebral Ischemia Reperfusion Injury Assessed using MALDI-MSI

**DOI:** 10.3390/metabo10010027

**Published:** 2020-01-07

**Authors:** Fang Tian, Runzhe Liu, Chaoxin Fan, Yi Sun, Xi Huang, Zongxiu Nie, Xin Zhao, Xiaoping Pu

**Affiliations:** 1 National Key Research Laboratory of Natural and Biomimetic Drugs, Peking University, Beijing 100191, China; 1611110483@pku.edu.cn (F.T.); 1110307320@pku.edu.cn (R.L.); fanchaoxin@bjmu.edu.cn (C.F.); sunyi@bjmu.edu.cn (Y.S.); zhaoxin2010@bjmu.edu.cn (X.Z.); 2 Department of Molecular and Cellular Pharmacology, School of Pharmaceutical Sciences, Peking University, Beijing 100191, China; 3 Key Laboratory of Analytical Chemistry for Living Biosystems, Institute of Chemistry Chinese Academy of Sciences, Beijing 100190, China; huangxi93@foxmail.com (X.H.); znie@iccas.ac.cn (Z.N.); 4 Beijing National Laboratory for Molecular Sciences, Beijing 100190, China

**Keywords:** cerebral ischemia reperfusion injury, thymoquinone, MALDI-MSI, small-molecule substance metabolism, energy metabolism

## Abstract

Thymoquinone is one of the main components present in *Nigella sativa* seeds and is known to have various biological functions in inflammation, oxidative stress, tumors, aging, and in lowering blood glucose levels. Few studies have focused on its neuroprotective effects and its regulation of small-molecule metabolites during cerebral ischemia reperfusion injury. In this study, transient middle cerebral occlusion (tMCAO) was used to establish the rat model of cerebral ischemia reperfusion injury. We investigated the effects of thymoquinone using matrix-assisted laser desorption ionization mass spectrometry imaging (MALDI-MSI) in a model of ischemia reperfusion injury to explore the changes in small-molecule metabolites in the brain. We found that that thymoquinone significantly improved neurobehavioral scores, reduced the cerebral infarct area, alleviated brain edema, and increased the number of normal neurons following injury. MALDI-MSI revealed that thymoquinone reduced abnormal accumulations of glucose, citric acid, succinate and potassium ions. Thymoquinone also increased the amount of energy-related molecules such as ADP, AMP, GMP, and creatine, antioxidants such as glutathione, ascorbic acid, and taurine, and other metabolism-related molecules such as glutamate, glutamine, aspartate, N-acetyl-L-aspartate, and sodium ions in damaged areas of the brain following cerebral ischemia reperfusion injury. In summary, based on the neuroprotective effect of thymoquinone on cerebral ischemia reperfusion injury, this study revealed the regulation of thymoquinone on energy metabolism and small-molecule substance metabolism.

## 1. Introduction

Stroke is one of the most severe causes of central nervous system disorders. Ischemic stroke accounts for a majority of strokes and is accompanied by high mortality, disability, and recurrence rates. Restoring the cerebral blood flow is imperative for recovery after ischemic stroke. However, restoring the blood flow causes a series of pathophysiological cascades that can lead to further damage to the brain, a process known as ischemia reperfusion injury [1].

*Nigella sativa* is a dicotyledonous plant belonging to the family Ranunculaceae and is mainly distributed in West Asia, the Middle East, Eastern Europe, and the Mediterranean countries [2]. Thymoquinone is the main bioactive ingredient in the seeds of *Nigella sativa* and has antioxidative, anti-inflammatory, and anti-infective effects. Using neurological dysfunction models, thymoquinone has been reported to have protective effects in stroke, schizophrenia, and epilepsy. Continuous administration of thymoquinone in pre-ischemia and reperfusion was shown to significantly reduce the number of necrotic neurons in the hippocampus [3]. In addition, thymoquinone mucoadhesive nanoemulsion (TMNE) significantly increased the in vivo bioavailability of thymoquinone and improved the spontaneous activity and forelimb grip strength in rats after middle cerebral artery occlusion [4]. Although these studies showed that thymoquinone can have a neuroprotective effect in cerebral ischemia reperfusion injury, the mechanism by which it does so has not yet been elucidated. Edaravone was a free radical scavenger with brain protection, which had been used clinically to improve neurological symptoms, daily activities and dysfunction caused by acute cerebral infarction. In the rat cerebral ischemia reperfusion model, edaravone had shown obvious neuroprotective effect, and was widely used as a positive drug for pharmacodynamics evaluation.

Matrix-assisted laser desorption ionization mass spectrometry imaging (MALDI-MSI) is a visual imaging technology that combines mass spectrometry and molecular imaging and can quickly and efficiently detect the spatial distribution and relative content of multiple molecules in tissues. To date, no studies have evaluated the effects of thymoquinone on small-molecule metabolites in the brain after ischemia–reperfusion injury using MALDI-MSI.

This study evaluated the effects of thymoquinone on cerebral ischemia–reperfusion injury in relation to neurological behavior scores, cerebral infarction area, brain edema injury, and histomorphology. Mass spectrometry imaging was used to further investigate the effects of thymoquinone on the metabolism of endogenous substances correlated with metabolism, energy metabolism, excitatory amino acids, phospholipids, antioxidant molecules, and metal ions after cerebral ischemia reperfusion injury. Analysis of small-molecule substance metabolism revealed the protective effects of thymoquinone in cerebral ischemia reperfusion injury.

## 2. Results

### 2.1. Neuroprotective Effects of Thymoquinone in the Cerebal Ischemia Reperfusion Injury Model

**Figure 1:**
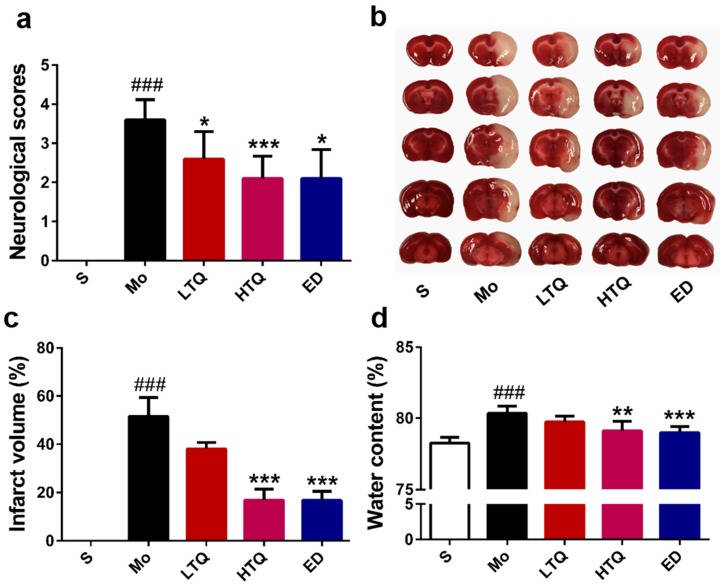
Protective effect of thymoquinone in a rat model of cerebral ischemia reperfusion injury. (**a**) Neurological deficit scores (*n* = 10), (**b**) Representative images of triphenyltetrazolium chloride-stained brain sections, (**c**) Infarct volume percentage (*n* = 6), (**d**) Brain water content (*n* = 6). Data are expressed as mean ± SD, ^###^ *p* < 0.001 vs. sham group; * *p* < 0.05, ** *p* < 0.01, *** *p* < 0.001 vs. model group. S: Sham; Mo: Model; LTQ: 2.5 mg/kg thymoquinone; HTQ: 5 mg/kg thymoquinone; ED: 3 mg/kg edaravone.

We used a modified 5-point Bederson scale to determine the effect of thymoquinone on the behavior of rats after cerebral ischemia–reperfusion injury. As shown in Figure 1a, the neurobehavioral performance in the sham group was as expected in untreated animals. Compared with the sham group, cerebral ischemia reperfusion injury significantly increased the neurobehavioral score (*p* < 0.001). Administration of 2.5 mg/kg (*p* < 0.05) or 5 mg/kg (*p* < 0.001) thymoquinone or edaravone (*p* < 0.05) significantly reduced the neurological deficits after cerebral ischemia–reperfusion injury.

Triphenyltetrazolium chloride (TTC) staining is shown in Figure 1b, where normal brain tissue is colored red and damaged brain areas after infarction are colored white. In the sham group, brain slices appeared with a bright red color, and no white-colored infarction areas were observed. Compared with the sham group, hemi-ischemic necrosis appeared on brain slices after cerebral ischemia–reperfusion injury, with the percentage of infarction being 51.52 ± 7.88% (*p* < 0.001) (Figure 1c). Thymoquinone (5 mg/kg) or edaravone significantly reduced the infarct size after injury, with percentages of hemi-cerebral infarction of 16.92 ± 4.49% (*p* < 0.001) and 16.76 ± 3.83% (*p* <0.001), respectively. The lower dose of thymoquinone (2.5 mg/kg) showed some improvement on the area of cerebral infarction, but this was not statistically significant.

After cerebral ischemia reperfusion injury, the damaged hemisphere was significantly edematous. Administration of 5 mg/kg thymoquinone or edaravone reduced the water content of the affected hemisphere (*p* < 0.01 for thymoquinone and *p* < 0.001 for edaravone) and alleviated edema damage caused by cerebral ischemia reperfusion injury (Figure 1d), whereas the lower dose of thymoquinone (2.5 mg/kg) did not show a significant improvement in the cerebral edema injury.

### 2.2. Histomorphological Changes Following Cerebral Ischemia–Reperfusion Injury

**Figure 2:**
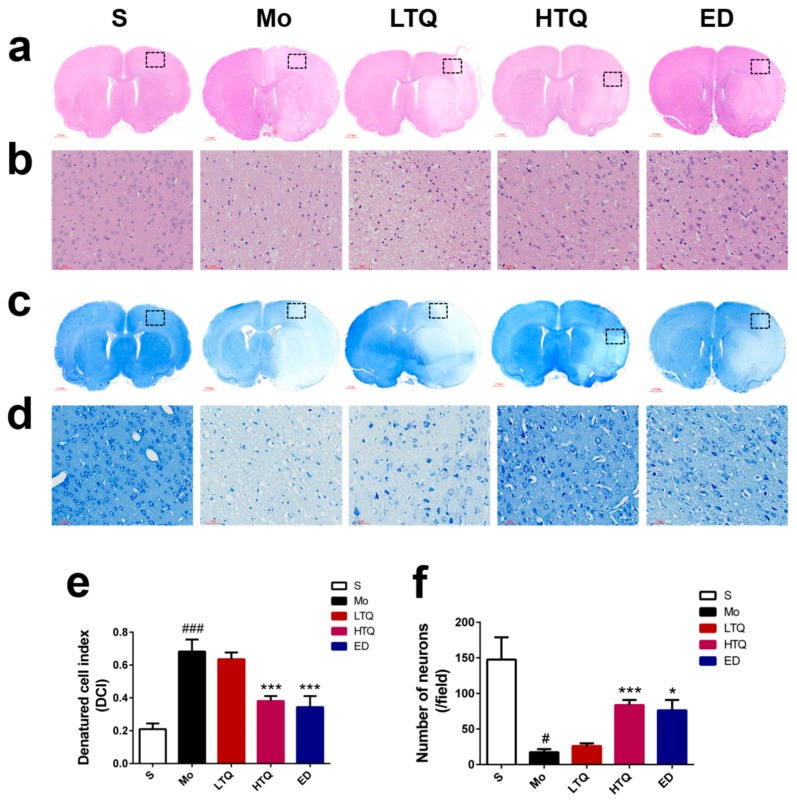
Effect of thymoquinone treatment on the morphology of cortical neuronal cells following cerebral ischemia reperfusion injury. (**a**,**b**) Representative photomicrographs of hematoxylin and eosin (HE) staining (5× and 200×). (**c**,**d**) Representative photomicrographs of Nissl staining (5× and 200×). (**e**) Quantification of denatured cells. (**f**) Quantification of Nissl-positive neurons. Black rectangular areas in the ischemic ipsilateral cortex of penumbra were amplified by automated microscopy and represent the regions selected for histology analysis. Data are expressed as mean ± SD (*n* = 4), ^#^ *p* < 0.05, ^###^ *p* < 0.001 vs. sham group; * *p* < 0.05, *** *p* < 0.001 vs. model group. S: Sham; Mo: Model; LTQ: 2.5 mg/kg thymoquinone; HTQ: 5 mg/kg thymoquinone; ED: 3 mg/kg edaravone.

Hematoxylin-eosin staining is typically used to evaluate the degree of nerve damage in the ischemic penumbra after cerebral ischemia reperfusion injury. As shown in Figure 2a,b, the cortical cells in the sham group were undamaged, presenting with clear cell boundaries, complete cell morphology, and a large number of cells. In contrast, severe cell damage occurred in the ischemic penumbra of the cortex in MCAO rats, with irregularly arranged cells, large numbers of vacuoles in the extracellular matrix, decreased cell numbers, altered cell morphology, pyknosis, and deep nuclear staining. Compared with the sham group, the number of degenerated cells in the cortical ischemic penumbra and the denatured cell index (DCI) increased significantly in the model group following cerebral ischemia–reperfusion injury (*p* < 0.001). Compared with the model group, administration of 5 mg/kg thymoquinone or edaravone significantly increased the number of normal cells in the ischemic penumbra and decreased DCI (*p* < 0.001 for thymoquinone and edaravone) (Figure 2e).

Nissl staining is typically used for the detection of Nissl bodies in the neuronal cytoplasm. An increase in the amount and size of Nissl bodies indicates a normal function of neuronal protein synthesis. However, when there is damage to the neurons, the number of Nissl bodies is either reduced or nonexistent. As shown in Figure 2c,d, sham neurons presented with normal morphology, with the nucleus located in the center of the cytoplasm, and with the cytoplasm containing large numbers of Nissl bodies. In contrast, neurons in the ischemic penumbra in the model group were significantly damaged, Nissl bodies were reduced in number and size, and the nuclei were pyknotic. Compared with the sham group, the staining intensity of Nissl bodies in the model group was decreased and the number of Nissl-positive neurons was significantly decreased (*p* < 0.05). Compared with the model group, 5 mg/kg thymoquinone or edaravone treatment significantly increased the number of surviving neurons in the ischemic penumbra per unit of field (*p* < 0.001 for thymoquinone and *p* < 0.05 for edaravone) (Figure 2f). However, 2.5 mg/kg thymoquinone treatment did not induce a significant change in the number of surviving neurons.

### 2.3. MALDI-MSI Analysis Following Cerebral Ischemia Reperfusion Injury

#### 2.3.1. Effect of Thymoquinone Treatment on Aerobic Oxidation-Related Small Molecules in the Damaged Brain

**Figure 3:**
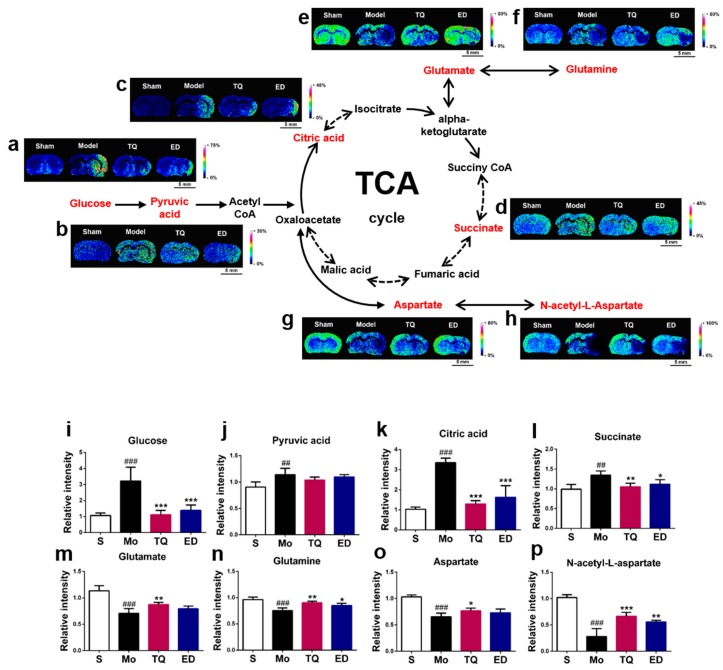
In situ matrix-assisted laser desorption ionization mass spectrometry imaging (MALDI-MSI) distribution and changes in aerobic oxidation-related molecules and amino acids. (**a**–**h**) Representative MALDI-MSI images of metabolites, (**i**–**p**) statistical results of the relative changes in metabolites, with the relative intensity of each metabolite calculated by the average intensity ratio of the ischemic hemisphere to the contralateral hemisphere. (**a**,**i**) Glucose, (**b**,**j**) Pyruvic acid, (**c**,**k**) Citric acid, (**d**,**l**) Succinate, (**e**,**m**) Glutamate, (**f**,**n**) Glutamine, (**g**,**o**) Aspartate, (**h**, **p**) N-acetyl-L-aspartate. The spatial resolution of MALDI-MSI images is 200 μm, and the bar scale is 5 mm. Data are expressed as mean ± SD (*n* = 4), ^##^ *p* < 0.01, ^###^ *p* < 0.001 vs. sham group; * *p* < 0.05, ** *p* < 0.01, *** *p* < 0.001 vs. model group. S: Sham; Mo: Model; TQ: 5 mg/kg thymoquinone; ED: 3 mg/kg edaravone.

Most cells obtain energy through aerobic oxidation, which is a primary mode of the glucose oxidation process. We detected mass spectrometry signals of four small molecules, including glucose, pyruvic acid, citric acid, and succinic acid, during aerobic oxidation. As shown in Figure 3, all four molecules were significantly increased (*p* < 0.01–0.001) after cerebral ischemia reperfusion injury. Prophylactic administration of 5 mg/kg thymoquinone significantly reduced the amount of glucose, citric acid, and succinic acid but not of pyruvate in the damaged areas of the brain (*p* < 0.01–0.001).

Through the tricarboxylic acid (TCA) cycle, the intermediate downstream metabolites of glucose α-ketoglutarate and oxaloacetate can also be transformed into the four excitatory neurotransmitters: Glutamate, glutamine, aspartate, and N-acetyl-L-aspartate. Mass spectrometry imaging results showed that cerebral ischemia reperfusion injury reduced the amount of all four neurotransmitters in the damaged brain areas (*p* < 0.001) (Figure 3). After administration of thymoquinone, the amount of glutamate, glutamine, N-acetyl-L-aspartate (*p* < 0.01–0.001) and aspartate (*p* < 0.05) were increased in the areas with brain damage. Figure S1 lists the partial mass spectrometry ion peaks for the aerobic oxidation process-related small molecules and neurotransmitter amino acids.

#### 2.3.2. Effect of Thymoquinone Treatment on Small Molecules Involved in Energy Metabolism in the Damaged Brain

**Figure 4:**
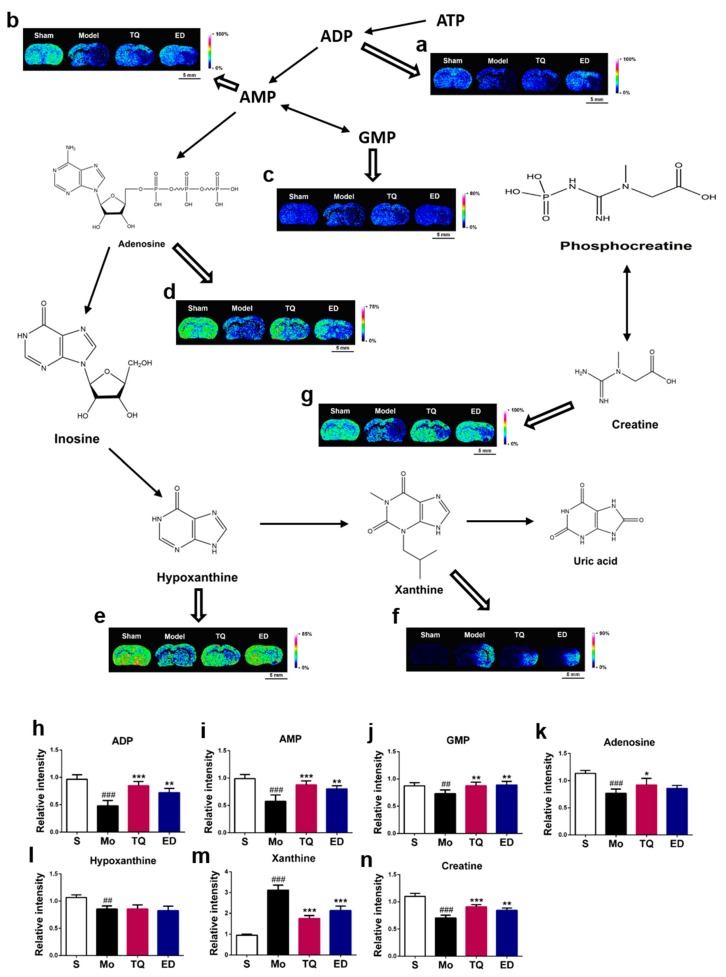
In situ matrix-assisted laser desorption ionization mass spectrometry imaging (MALDI-MSI) distribution and changes in energy metabolism-related small molecules. (**a**–**g**) Representative MALDI-MSI images of metabolites, (**h**–**n**) statistical results of the relative changes in metabolites, with the relative intensity of each metabolite calculated by the average intensity ratio of the ischemic hemisphere to the contralateral hemisphere. (**a**,**h**) ADP, (**b**,**i**) AMP, (**c**,**j**) GMP, (**d**,**k**) Adenosine, (**e**,**l**) Hypoxanthine, (**f**,**m**) Xanthine, (**g**,**n**) Creatine. The spatial resolution of MALDI-MSI images is 200 μm, and the bar scale is 5 mm. Data are expressed as mean ± SD (*n* = 4), ^##^ *p* < 0.01, ^###^ *p* < 0.001 vs. sham group; * *p* < 0.05, ** *p* < 0.01, *** *p* < 0.001 vs. model group. S: Sham; Mo: Model; TQ: 5 mg/kg thymoquinone; ED: 3 mg/kg edaravone.

Cerebral ischemia reperfusion injury breaks the normal energy metabolism process. ATP is a form of energy that is directly utilized by cells. Using 1,5-DAN hydrochloride-assisted MSI, we detected changes in the ATP downstream energy metabolites in the damaged areas of the brain (Figure 4). We found that cerebral ischemia reperfusion injury reduced the levels of five ATP downstream metabolites (ADP, AMP, GMP, adenosine, and hypoxanthine) and creatine in the damaged brain (*p* < 0.01–0.001). The xanthine level was also significantly increased (*p* < 0.001). We found that thymoquinone have a regulatory effect on several energy metabolism-related small molecules during cerebral ischemia reperfusion injury, and MSI results showed that it induced increases in ADP, AMP, GMP, creatine (*p* < 0.01–0.001) and adenosine (*p* < 0.05) levels in the damaged brain. The xanthine level was decreased (*p* < 0.001), but no statistical difference was noted for hypoxanthine. Figure S2 lists the partial mass spectrometry ion peaks for energy metabolism-related small molecules.

#### 2.3.3. Effect of Thymoquinone Treatment on Lipid Molecules in the Damaged Brain

**Figure 5:**
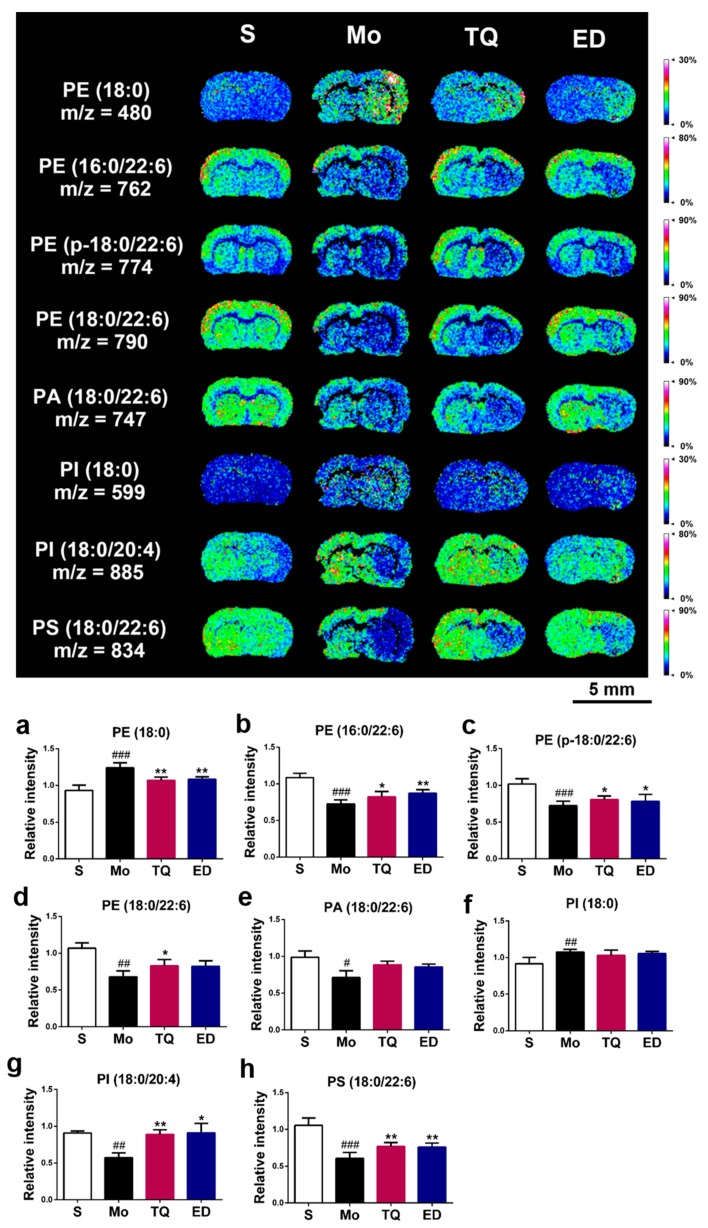
In situ matrix-assisted laser desorption ionization mass spectrometry imaging (MALDI-MSI) distribution and change in phospholipid molecules. (**a**–**h**) Statistical results of the relative changes in metabolites, with the relative intensity of each metabolite calculated by the average intensity ratio of the ischemic hemisphere to the contralateral hemisphere. (**a**) PE (18:0), (**b**) PE (16:0/22:6), (**c**) PE (p-18:0/22:6), (**d**) PE (18:0/22:6), (**e**) PA (18:0/22:6), (**f**) PI (18:0), (**g**) PI (18:0/20:4), (**h**) PS (18:0/22:6). The spatial resolution of MALDI-MSI images is 200 μm, and the bar scale is 5 mm. Data are expressed as mean ± SD (*n* = 4), ^#^ *p* < 0.05, ^##^ *p* < 0.01, ^###^ *p* < 0.001 vs. sham group; * *p* < 0.05, ** *p* < 0.01 vs. model group. S: Sham; Mo: Model; TQ: 5 mg/kg thymoquinone; ED: 3 mg/kg edaravone.

Phospholipid molecules of various molecular weights can also be detected using 1,5-DAN hydrochloride as a matrix. Figure 5 shows the spatial distribution and relative content of eight altered phospholipid molecules in the areas with brain damage. After cerebral ischemia reperfusion injury, the levels of phosphatidylethanolamine (PE), (16:0/22:6), PE (*p*-18:0/22:6), PE (18:0/22:6), phosphatidylinositol (PI) (18:0/20:4), phosphatidylserine (PS), (18:0/22:6) (*p* < 0.01–0.001) and phosphatidic acid (PA), (18:0/22:6) (*p* < 0.05) were significantly decreased, whereas the levels of PE (18:0) and PI (18:0) were significantly increased (*p* < 0.01–0.001). Thymoquinone had a regulatory effect on the levels of small molecules, such as PI (18:0/20:4), PS (18:0/22:6), PE (18:0) (*p* < 0.01), PE (16:0/22:6), PE (p-18:0/22:6), and PE (18:0/22:6) (*p* < 0.05). No statistical difference was found for PA (18:0/22:6) and PI (18:0) after thymoquinone administration. Figure S3 lists the partial mass spectrometry ion peaks for phospholipid molecules.

#### 2.3.4. Effect of Thymoquinone Treatment on Metal Ions, Antioxidant Molecules, and Other Metabolic Small Molecules in the Damaged Brain

**Figure 6:**
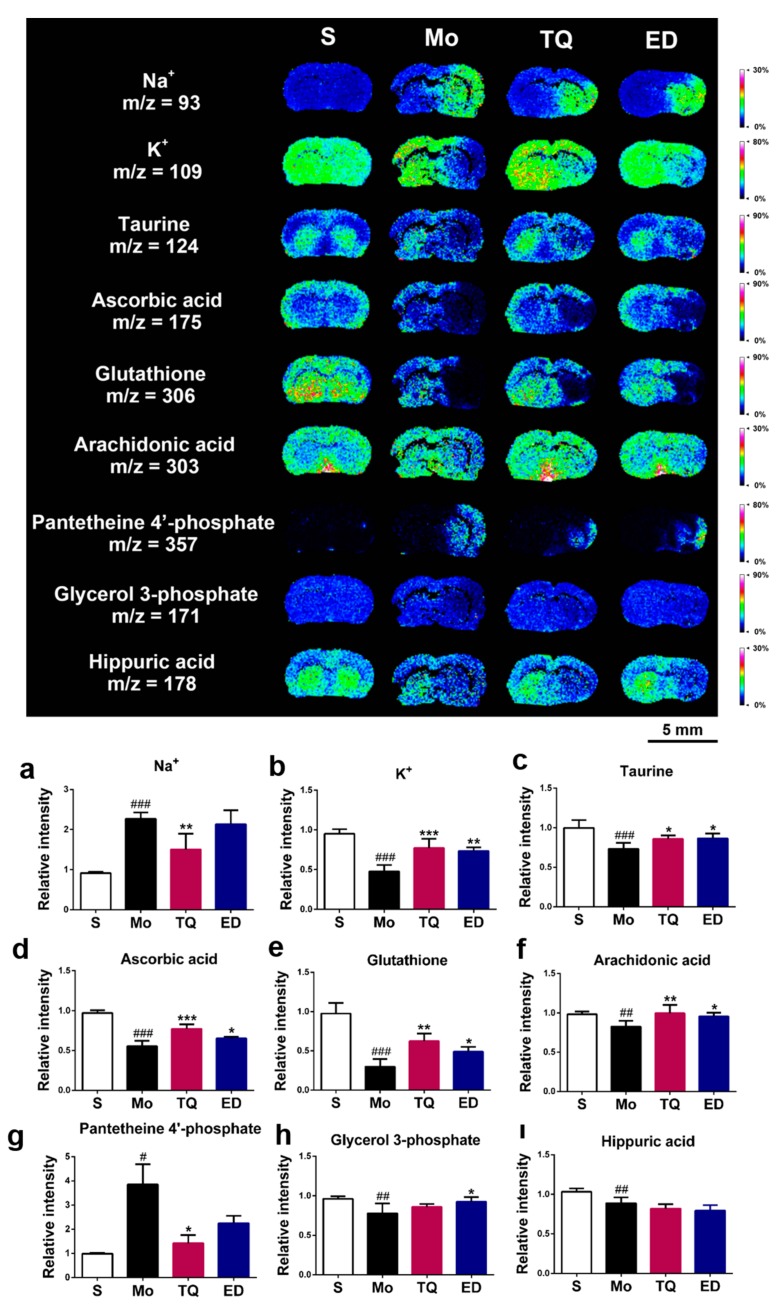
In situ matrix-assisted laser desorption ionization mass spectrometry imaging (MALDI-MSI) distribution and change in other metabolic molecules. (**a**–**i**) Statistical results of the relative changes in metabolites, with the relative intensity of each metabolite calculated by the average intensity ratio of the ischemic hemisphere to the contralateral hemisphere. (**a**) Na^+^, (**b**) K^+^, (**c**) Taurine, (**d**) Ascorbic acid, (**e**) Glutathione, (**f**) Arachidonic acid, (**g**) Pantetheine 4′-phosphate, (**h**) Glycerol 3-phosphate, (**i**) Hippuric acid. The spatial resolution of MALDI-MSI images is 200 μm, and the bar scale is 5 mm. Data are expressed as mean ± SD (*n* = 4), ^#^ *p* < 0.05, ^##^ *p* < 0.01, ^###^ *p* < 0.001 vs. sham group; * *p* < 0.05, ** *p* < 0.01, *** *p* < 0.001 vs. model group. S: Sham; Mo: Model; TQ: 5 mg/kg thymoquinone; ED: 3 mg/kg edaravone.

We also found changes in the damaged areas of the brain in terms of sodium and potassium ions. As shown in Figure 6a,b, cerebral ischemia reperfusion injury disrupted the steady-state equilibrium of normal ions, exhibiting an increase in sodium ion levels (*p* < 0.001) and decrease in potassium ion levels (*p* < 0.001) in the damaged areas of the brain. Thymoquinone had a significant regulatory effect on the levels of the two metal ions, reducing the sodium ion level and increasing the potassium ion level (*p* < 0.01–0.001).

In the pathological process of cerebral ischemia reperfusion injury, excessive accumulation of reactive oxygen species (ROS) can mediate the oxidative stress response in the body, and the antioxidant system in the body can remove excess ROS to protect it from excessive oxidative damage. We used mass spectrometry imaging to investigate the changes in the levels of three antioxidant molecules: Taurine, ascorbic acid, and reduced glutathione. As shown in Figure 6c–e, the levels of these three antioxidant molecules in the damaged areas of the brain in the model group were significantly lower (*p* < 0.001) than those in the sham group. Compared with the model group, the levels of ascorbic acid, reduced glutathione (*p* < 0.01–0.001), and taurine (*p* < 0.05) were significantly increased after thymoquinone administration.

In addition, we found four small molecules (arachidonic acid, pantetheine 4′-phosphate, glycerol 3-phosphate, and hippuric acid) that showed changes in the areas with brain damage. The mass spectrometry imaging results in Figure 6f–i show that the levels of arachidonic acid, glycerol 3-phosphate, and hippuric acid in the injured brain were significantly decreased after cerebral ischemia–reperfusion injury (*p* < 0.01), whereas the level of pantetheine 4′-phosphate increased (*p* < 0.05). Compared with the model group, thymoquinone administration significantly reduced the levels of pantetheine 4′-phosphate content (*p* < 0.05) and increased the content of arachidonic acid (*p* < 0.01). However, no significant changes were found for glycerol 3-phosphate and hippuric acid. Figure S4 lists the partial mass spectrometry ion peaks for other metabolic small molecules.

#### 2.3.5. Segmentation Analysis

**Figure 7:**
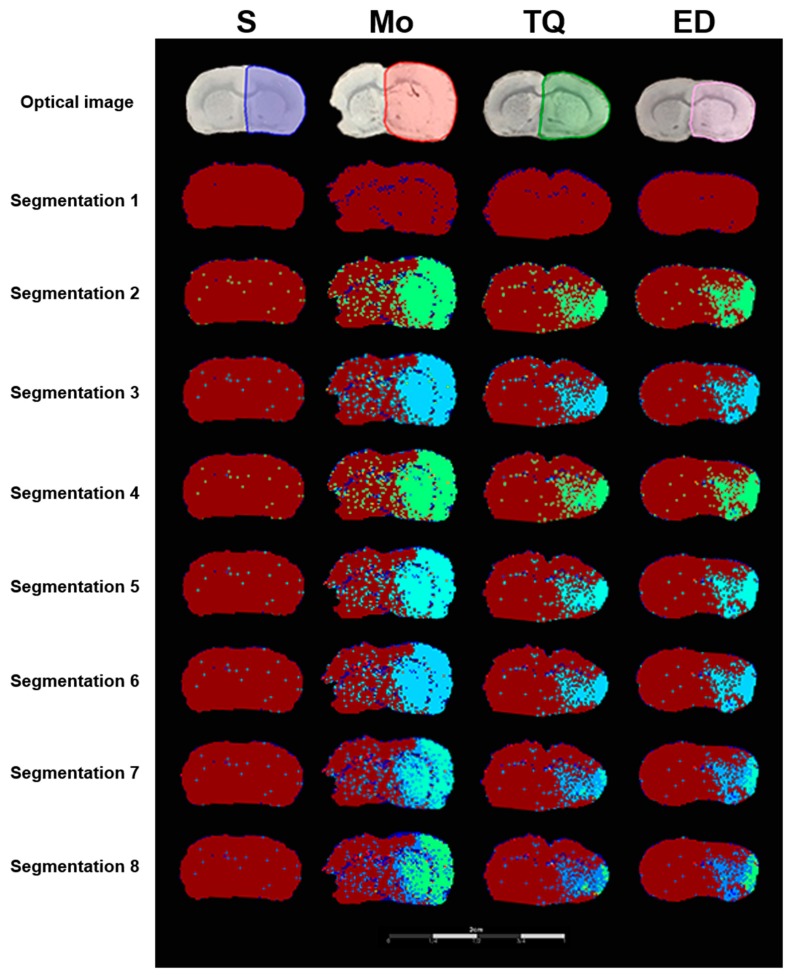
Representative segmented cluster images of brain sections obtained using the SCiLS Lab software. Optical images represent the scanning image of the brain sections with the sprayed matrix. Regions of interest (ROI) were selected using different colors for MALDI semi-quantitative analysis. Segmentation 1 to 8 separated the ischemic regions and normal regions, and showed the degree of damage within ischemic regions. Cluster analysis separated the ischemia-damaged regions from normal regions, with different grades of cluster analysis further dividing the ischemic area. S: Sham; Mo: Model; TQ: 5 mg/kg thymoquinone; ED: 3 mg/kg edaravone.

Mass spectrometry imaging can effectively combine molecular imaging and mass spectrometry analysis to display the spatial distribution and relative content of molecules with different *m*/*z*. The results of MSI segmentation analysis showed that cerebral ischemia reperfusion injury damaged the right hemisphere mainly in the cortex and striatum. Further segmentation analysis (Figure 7, segmentation 2 to segmentation 8) showed that the ischemic hemispheres in the model group can be divided into normal brain regions, ischemic penumbra regions, and ischemic core regions. As shown in segmentation 8 of Figure 7, the right ischemic area showed different colors, with dark red representing the normal brain regions, blue representing the ischemic penumbra regions and green representing the ischemic core regions. Compared with the sham group, the model group showed ischemic injury on the right hemisphere, with a larger ischemic core area and the ischemic penumbra region outside the ischemic core region. Thymoquinone administration reduced the area of ischemic penumbra regions and ischemic core regions. The results of the segmentation analysis were consistent with the results of the TTC and histomorphometric staining.

## 3. Discussion

**Figure 8:**
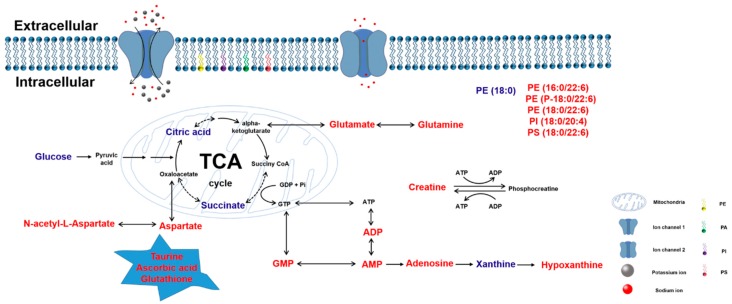
Summary of changes in endogenous small molecules as detected by MALDI-MSI. Small molecules that were significantly increased in ischemia-damaged regions after thymoquinone administration are represented in red; those that were significantly decreased are represented in blue.

Cerebral ischemia reperfusion injury is the restoration of blood flow after vascular occlusion, which results in further damage to the brain tissues. In this study, we used the transient middle cerebral artery occlusion (tMCAO) model to simulate cerebral ischemia reperfusion injury. Figure 8 summarizes the effects of thymoquinone on small-molecule metabolism. We found that thymoquinone had a protective effect on cerebral ischemia–reperfusion injury by decreasing the neurological deficit scores, reducing the brain infarct size, alleviating brain edema damage, improving cell morphology damage, and increasing the number of normal neurons. MALDI-MSI analysis indicated that thymoquinone was able to regulate the abnormal metabolism in the areas with brain damage by promoting glucose aerobic oxidation, regulating intracellular energy metabolism, improving phospholipid molecular levels, increasing the content of antioxidant small molecules, and balancing the homeostasis of sodium and potassium ions. These regulatory effects of thymoquinone ultimately reduced the damage in the brain tissue.

In this study, the common and recognized rats model of cerebral injury was established by ischemia for 2 h and reperfusion for 24 h [5,6]. The time of ischemia and reperfusion is different in different animal species. The duration of ischemia and reperfusion was mainly based on the physiological characteristics of rats. The purpose of this study was to investigate the effect of cerebral injury on small molecule metabolism, so samples were collected after 2 h of ischemia and 24 h of reperfusion. However, this sampling timeline may affect the determination of some small molecules. With the prolonged reperfusion time after ischemia, the animal’s physiopathological state may enter the recovery period, and some small metabolic molecules may return to baseline levels. Therefore, we took the samples immediately after 24 h reperfusion to reduce the bias in metabolite determination.

As the main active ingredient of *Nigella sativa*, thymoquinone has a wide range of neuropharmacological functions with reported protective effects against various central nervous system diseases. Thymoquinone was found to interact with Aβ_1_ to prevent its accumulation and slow the development of Alzheimer’s disease [7]. In a rotenone-induced Parkinson’s disease model, thymoquinone was shown to alleviate mitochondrial dysfunction and oxidative stress to protect the dopaminergic neurons [8]. Thymoquinone also enhanced the memory and exerted antipsychotic effects via the reduction of dopamine levels and acetylcholinesterase activity and the increase of glutathione levels [9]. In addition, in a model of transient forebrain ischemia, thymoquinone-loaded nanoparticles were shown to alleviate hippocampal damage caused by ischemia [8]. Thymoquinone-loaded PLGA–chitosan nanoparticles via nose-to-brain administration were able to reduce the infarct volume in rat brains after cerebral ischemia reperfusion, and the locomotory activity and forelimb grip strength were also subsequently increased. Thymoquinone reduced lipid peroxidation in the brain after middle cerebral artery occlusion and increased the glutathione level and the activity of enzymes such as catalase and superoxide dismutase [10]. Similarly, Hosseinzadeh et al. [11] used a model of transient global cerebral ischemia induced by a four-vessel-occlusion method to show that thymoquinone played a protective role by reducing the levels of malondialdehyde in the hippocampus after cerebral ischemia reperfusion and inhibiting lipid peroxidation.

This study showed that prophylactic administration of 5 mg/kg thymoquinone significantly improved the neurobehavioral scores and decreased the percentage of infarction and water content in the brain after ischemia–reperfusion injury. Thymoquinone was able to improve ischemic penumbra by reducing the number of degenerated cells, increasing the number of Nissl-positive neurons, decreasing the infarct area, and exerting neuroprotective effects. Compared with edaravone, thymoquinone displayed a better neuroprotective effect in improving neurobehavioral scores and increasing the number of Nissl-positive neurons. The lower dose of 2.5 mg/kg led to an improved performance in the neurological deficit test but limited effects on the other indicators.

Based on the neuroprotective functions, we further investigated the effects of thymoquinone on endogenous small-molecule metabolism in the brain tissues after cerebral ischemia reperfusion injury using mass spectrometry imaging combined with a 1,5-DAN hydrochloride matrix.

The aerobic oxidation of glucose is the primary means by which most cells derive energy and include the glycolytic pathway, pyruvate oxidative decarboxylation, the TCA cycle, and oxidative phosphorylation. As the main energy-consuming organ, the brain is highly sensitive to ischemia and hypoxia. Occlusion of the middle cerebral artery can result in a scarce supply of oxygen and glucose, leading to the destruction of the aerobic oxidation process [12]. Mass spectrometry imaging results in this study showed that glucose, pyruvate, citric acid, and succinate were abnormally accumulated in the damaged brain after cerebral ischemia reperfusion injury, which is consistent with the findings by Huang et al. [13]. However, in their study, the level of succinate, the TCA cycle intermediate, was significantly lower in the permanent middle cerebral artery occlusion (pMCAO) group than in the sham group. This finding was contradictory to our result obtained by MSI, and this may have been due to differences between permanent and transient MCAO. We found that the content of glucose, citric acid, and succinate in the damaged brain area increased after thymoquinone administration, and this improvement was also observed in edaravonetreatment group. Currently, there are no reports on the effect of thymoquinone on aerobic oxidation process. We used mass spectrometry imaging for the first time to reveal the correlation between thymoquinone and aerobic oxidation-related molecules. Thanks to the antioxidant biological activity, thymoquinone can act as a redox mediator to taking the “excess” of electrons from the respiratory complexes during reperfusion.

The TCA cycle is an important pathway that links glucose and amino acids. The TCA cycle intermediate α-ketoglutarate can be converted into glutamate, and the mutual conversion between the two neurotransmitters is achieved through the glutamate–glutamine cycle. As an important neurotransmitter in the central nervous system, excess extracellular glutamate can overstimulate glutamate receptors, leading to excitotoxicity [14]. Excitotoxicity has been described in various neurological diseases, such as Alzheimer’s disease, Parkinson’s disease, cerebral ischemia, and epilepsy.

N-acetyl-L-aspartate, a small amino acid found in high amounts in the brain, is mainly synthesized and stored by neurons and regulated by oligodendrocytes and can be enzymatically interconverted into aspartate. Upon occlusion of the middle cerebral artery, the glutamate and N-acetyl-L-aspartate levels in the affected brain areas decreased, whereas the glutamine level was significantly increased [13]. Proton nuclear magnetic resonance spectroscopy revealed that N-acetyl-L-aspartate was present in the neurons in normal brain tissue at a higher concentration but that was significantly reduced in pathological conditions such as impaired cellular metabolism and neuronal damage [15,16]. Magnetic resonance spectroscopy showed a significant reduction in N-acetyl-L-aspartate levels in the infarct region of patients after ischemic stroke [17]. Consistent with the findings of these studies, we found a decrease in glutamate, N-acetyl-L-aspartate, and aspartate levels after cerebral ischemia–reperfusion injury by MSI.

There are few reports on the regulation of the four excitatory amino acids by thymoquinone. Thymoquinone was shown to modulate glutamate-mediated neurotoxicity and inhibit cell apoptosis and Aβ formation through neuromodulation [18]. This study was the first to use mass spectrometry imaging to explore the function of thymoquinone on excitatory amino acids after cerebral ischemia–reperfusion injury. Compared with edaravone, thymoquinone could not only increase the levels of glutamine and N-acetyl-L-aspartate after injury but also increase the levels of glutamate and aspartate, and this increased the understanding of its protective mechanism in cerebral ischemia–reperfusion injury.

Mitochondria are the regulatory centers of cellular energy metabolism and oxidative stress. Mitochondrial oxidative phosphorylation is the main pathway for ATP production. The creatine–phosphocreatine system plays a significant role in the transport, storage, and utilization of high-energy phosphate bonds in the body. After cerebral ischemia reperfusion injury, the normal electron transport processes in the mitochondrial oxidative respiratory chain are inhibited, hindering oxidative phosphorylation in the mitochondria [19]. Cerebral ischemia reperfusion injury can also lead to the excessive accumulation of ROS, which can further aggravate mitochondrial damage and degrade cellular energy metabolism [20]. In this study, mass spectrometry imaging was used to investigate the changes in energy-related small molecules in the areas with brain damage. Except for xanthine, most of the downstream ATP metabolites and creatine in the damaged brain were decreased. Farooqui et al. [21] previously found that thymoquinone could regulate the metabolism of carbohydrates in the kidneys by improving the activity of enzymes and also alleviate the effect of cisplatin-induced toxicity on the intracellular energy metabolism. Similarly, Shahid et al. [22] showed the effect of thymoquinone on cisplatin-induced energy depletion in intestinal cells. There is no report on the regulation of ATP and related endogenous small molecules by thymoquinone, and our results showed that thymoquinone and positive control (edaravone group) possess the comparable ability in regulating energy-related small molecules. This study showed for the first time that thymoquinone can regulate energy-related small molecules after cerebral ischemia reperfusion injury.

Phospholipids are the basic structural components of neuronal cell membranes and play an important role in the maintenance of normal cell morphology and physiological functions. Shanta et al. [23] used MALDI MS to show the differential expression of various phospholipid molecules in both the ischemic and normal regions of the brain in an ischemic model. They identified 11 upregulated phospholipids, including lysophosphatidylcholine (LPC), phosphatidylcholine (PC), and sodiated forms of sphingomyelin (SM) and PCs, as well as 7 downregulated phospholipids in the areas with ischemic damage. Nielsen et al. [24] used desorption electrospray ionization and MALDI to study the expression of different phospholipid molecules in the brain at different time points after pMCAO. In this study, 1,5-DAN hydrochloride solution was used as a matrix, and mass spectrometry imaging was used to investigate changes in four phospholipid molecules (PE, PA, PI, and PS) in the ischemic brain. Cerebral ischemia–reperfusion injury reduced the levels of PE (16:0/22:6), PE (p-18:0/22:6), PE (18:0/22:6), PA (18:0)/22:6), PI (18:0/20:4), and PS (18:0/22:6) and increased the levels of PE (18:0) and PI (18:0). Thymoquinone increased the levels of PE (16:0/22:6), PE (p-18:0/22:6), PE (18:0/22:6), PI (18:0/20:4), and PS (18:0/22:6) and decreased the level of PE (18:0), thereby regulating phospholipid levels in areas with brain damage. Thymoquinone and edaravone had their own characteristics in the regulation of phospholipid molecules, and better improvement was observed in PE (18:0/22:6), PI (18:0/20:4), and PS (18:0/22:6) in thymoquinone-treatment group. We showed for the first time the protective effect of thymoquinone on cerebral ischemia–reperfusion injury through regulation of phospholipid metabolism.

Sodium and potassium ions ensure the homeostasis of the intracellular environment under physiological conditions and maintain the normal electrophysiological function of neurons. The energy supply to the brain after ischemia is converted from aerobic to anaerobic glycolysis. Excessive accumulation of lactic acid causes lactic acidosis, and accumulation of lactate/H^+^ activates the Na^+^/H^+^ exchanger and increases the amount of Na^+^ in the cells [13]. Liu et al. [25] used mass spectrometry imaging to investigate the effect of butylphthalide after cerebral ischemia infarction and found that the Na^+^ content in the injured hemisphere was increased, whereas the K^+^ content was decreased after pMCAO. This data is consistent with our findings after cerebral ischemia–reperfusion injury. However, studies on the regulation of metal ions by thymoquinone have not been reported. We found that thymoquinone had a better effect in homeostasis. Thymoquinone reduced the content of sodium ions and increased the content of potassium ions, while edaravone only improved the potassium ions.

Oxidative stress caused by abnormal accumulation of ROS is another major pathological process of cerebral ischemia–reperfusion injury. Cerebral ischemia reperfusion injury increases the electron leakage in the oxidative respiratory chain and promotes the production of ROS. Excess ROS in mitochondria can consume in vivo antioxidants and inhibit the endogenous antioxidant defense system. In this study, mass spectrometry imaging was used to detect the mass spectrometric signals of three endogenous antioxidants: Taurine, ascorbic acid, and reduced glutathione. The levels of these antioxidants were significantly reduced in the areas with brain damage after cerebral ischemia–reperfusion injury. Reduced glutathione and ascorbic acid are important antioxidants in the body that directly remove ROS from the brain. Taurine exerts an antioxidant effect by inhibiting ROS production and the inflammatory response through the mitochondria electron transport chain [26]. Studies have reported the antioxidative effects of thymoquinone. Thymoquinone was shown to reduce the levels of malondialdehyde and protect against lipid peroxidation damage caused by cerebral ischemia–reperfusion [11]. Xiao et al. [10] showed that thymoquinone could reduce lipid peroxidation in the brain after middle cerebral artery occlusion and increase the glutathione level and the activity of catalase and superoxide dismutase. However, the effects of thymoquinone on taurine and ascorbic acid have not yet been reported. This study shed light on the regulation of reduced glutathione by thymoquinone and found that thymoquinone could increase the level of these two antioxidant molecules in the ischemic areas of the brain.

Finally, mass spectrometry imaging showed changes in arachidonic acid, pantetheine 4′-phosphate, glycerol 3-phosphate, and hippuric acid. Thymoquinone increased the level of arachidonic acid and reduced the level of pantetheine 4′-phosphate but had no effect on glycerol 3-phosphate and hippuric acid levels, while edaravone could only improve the level of glycerol 3-phosphate. Arachidonic acid is a major component of the biomembrane structure and is released from the membrane structure and converted into various physiologically active lipid molecules under catalysis by phospholipase A2. In addition, arachidonic acid plays an active role in synaptic plasticity, membrane fluidity, and neurogenesis [27]. We hypothesize that the regulation of arachidonic acid by thymoquinone may be related to the anti-oxidant and anti-inflammatory effects of thymoquinone by regulating the amount of ROS to reduce lipid peroxidation damage by ROS in the biomembrane structure. At present, there are few reports on the relationship between pantetheine 4′-phosphate and nerve damage. Our results suggest that cerebral ischemia–reperfusion injury leads to an increase in pantetheine 4′-phosphate levels, but the specific mechanisms by which this occurs requires further research.

## 4. Materials and Methods

### 4.1. Chemicals and Reagents

**Figure 9:**
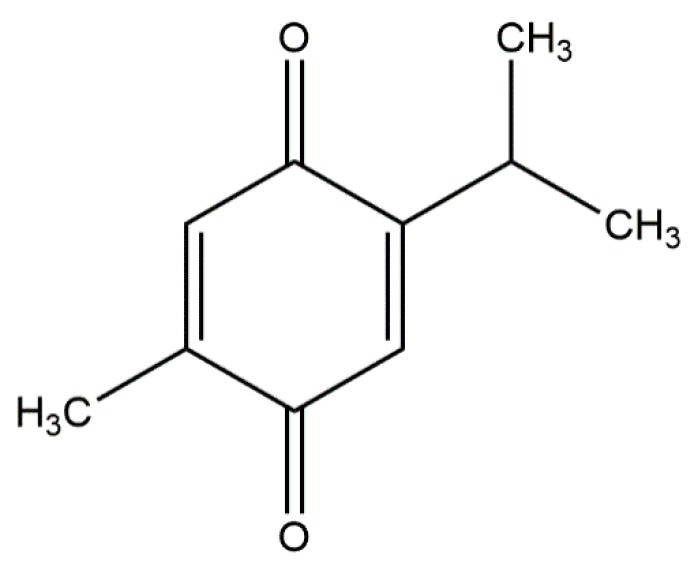
Chemical structure of thymoquinone.

Thymoquinone (Figure 9), 2,3,5-triphenyltetrazolium chloride, and 1,5-diaminonaphthalene were purchased from Sigma-Aldrich (St. Louis, MO, USA). Edaravone was purchased from Jiangsu Simcere Pharmaceutical Co., Ltd. (Nanjing, China).

### 4.2. Experimental Animals

SPF 6–7-week-old (210–230 g) male Sprague–Dawley rats were purchased from Beijing Huafukang Biotechnology Co., Ltd. (animal certificate number: SCXK (Beijing) 2014-0004). The animals were housed in an SPF environment in the Animal Department of Peking University at a temperature of 22 °C–24 °C, humidity of 50–60%, and a 12 h light/12 h dark cycle, with free access to standard rodent diet and drinking water. Animal experiments were conducted in accordance with the principles of the NIH Guide for the Care and Use of Laboratory Animals and approved by the Experimentation Ethics Committee on Animal Use of the College of Medicine, Peking University (approval number: LA2016159; date of approval: March 1st, 2016).

### 4.3. Cerebral Ischemia–Reperfusion Model

A cerebral ischemia reperfusion model was established using the tMCAO method [28], i.e., reperfusion for 24 h following ischemia for 2 h. Rats were intraperitoneally injected with 60 mg/kg pentobarbital sodium, following which the right common carotid artery, external carotid artery, and internal carotid artery were exposed. The silicone-coated MCAO filament (wire diameter: 0.28 mm; head diameter: 0.38 ± 0.02 mm; and wire length: 45 mm) was inserted into the internal carotid artery through the external carotid artery incision and was directed to the start point of the anterior artery to block the blood flow to the middle cerebral artery. After 2 h, the filament was retrieved to restore the blood flow, and the skin was then sutured. The sham group underwent a similar surgery without insertion of the filament. The body temperature was maintained at 37 ± 1°C throughout the surgery.

Rats were randomized into the sham group, model group, thymoquinone pre-treatment group (2.5 mg/kg and 5 mg/kg), and edaravone pre-treatment group (positive control drug, at 3 mg/kg), with 15 rats per group. Thymoquinone was solubilized in olive oil [29] and was administered intraperitoneally at 0.5 mL/100 g body weight qd for 7 days. MCAO surgery was performed 30 min after the final administration. The success rate of this model was 85%. The sham group and the model group received vehicle treatment only.

### 4.4. Animal Behavioral Test

Following establishment of the cerebral ischemia reperfusion model, the behavioral scores were obtained using modified Bederson scores, as previously described [30]. The specific scoring criteria were as follows: 0, no deficit; 1, forelimb flexion deficit on the contralateral side; 2, decreased resistance to lateral push and torso turning to the ipsilateral side when held by the tail; 3, significant circling and reduced capability to bear weight on the affected side; and 4, animal rarely moving spontaneously.

### 4.5. Infarct Volume and Edema Damage Assessment

Rats were anesthetized with an intraperitoneal injection of pentobarbital sodium (60 mg/kg) [31]. Brain tissue was collected and frozen at −20 °C for 30 min, and 2 mm brain slices [32] of the coronal plane were prepared and placed in 2% TTC/phosphate-buffered saline (PBS) solution and incubated at 37 °C for 15 min in the dark. The infarction area, the area of intact ipsilateral hemisphere, and the area of intact contralateral hemisphere were calculated for each brain slice using Image J software. To correct for the confounding effect of cerebral edema on the percentage of infarction, the following formula was used: percentage of infarction (%) = [total area of infarction − (total area of intact ipsilateral hemisphere − total area of intact contralateral hemisphere)]/total area of intact contralateral hemisphere] × 100, as described previously [33].

To assess the edema damage, we determined the ratio of the water content in the brain tissue and the total weight of the brain [32]. Briefly, the weight of the brain tissue was measured after TTC staining. The brain tissue was then placed at 110 °C for 16 h for drying and weighed again. The water content of the brain tissues was calculated using the following formula: Water content (%) = [(wet weight of brain tissue − dry weight of brain tissue)/brain tissue wet weight] × 100.

### 4.6. Analysis of Histomorphological Features

After establishment of the cerebral ischemia reperfusion model, four rats were randomly selected from each group for hematoxylin and eosin (HE) staining and Nissl staining, as described previously [34,35]. Rats were terminally anesthetized with an intraperitoneal injection of pentobarbital sodium (60 mg/kg) and perfused with normal saline and 4% paraformaldehyde/PBS solution through the heart. Whole brain tissue was collected and immersed in a 4% paraformaldehyde/PBS solution for 24 h at 4 °C. Brain tissues from bregma at 0.3 mm to −1.8 mm [34] were embedded in paraffin and prepared into 4-μm coronal sections. Tissue sections were scanned using a Motic BA600 automated microscope and photographed using Motic DS Assistant Lite software at 5× and 200× magnifications. At the 200× magnification, four non-coinciding fields were randomly selected for quantitative analysis in the ischemic penumbra of the right hemisphere. The DCI [36] was calculated using HE images to indicate the degree of nerve damage using the following formula: DCI = (number of degenerated cells/total number of cells) × 100. The number of viable neurons containing Nissl bodies was counted per unit of field of view [37].

### 4.7. Preparation of Samples for Mass Spectrometry Imaging

Brain tissues were isolated, and 10-μm coronal brain slices were prepared on a cryostat (Leica CM1950, Leica Microsystems). The ITO slides carrying the brain slices were dried in a vacuum at room temperature for 1 h to remove moisture. A 1,5-DAN hydrochloride solution was prepared as a spray matrix for mass spectrometry imaging according to the method described by Liu et al. [38]. Brain slices were sprayed with a small-sized humidifier nozzle. The parameters of the humidifier were as follows: Working voltage, 5 V; working current, 400 MA; power, 2 W; spray speed, 35 mL/h. Specific spraying experiments were in accordance with the method described by Huang et al. [39].

### 4.8. Mass Spectrometry Imaging

Mass spectrometry imaging experiments were performed using the Autoflex Speed^TM^ MALDI-TOF/TOF MS (Bruker Daltonics, Bremen, Germany). In the reflection negative ion mode, the spatial distribution and relative content of small molecular substances, with an *m*/*z* ranging from 60–1000 Da, were collected with a resolution of 200 μm. The operating parameters of the Autoflex mass spectrometer were as follows: Laser frequency, 2000 Hz; reflector pulse ion extraction time, 80 ns; acceleration voltage, 20.00 kV; extraction voltage, 17.90 kV; lens voltage, 5.85 kV; reflector voltage, 21.15 kV. At the end of the imaging session and according to the method described by Liu et al. [38,40], the identified small molecules were used to obtain mass spectrometry images of different *m*/*z* small molecules using the FlexImaging software. The SCiLS Lab software was used to calculate the average ion intensity of the left and right hemispheres and the ratio of the average ion intensity of the right hemisphere to that of the left hemisphere, indicating the relative content of different small molecules.

### 4.9. Segmentation Analysis

After completion of the mass spectrometry imaging, the results were imported into the SCiLS Lab software, and “Segmentation” was selected under the “Pipelines” item. According to the calculation method of “Segmentation,” if the tissue mass spectrum is similar, the composition and structure of the tissue are considered to be consistent, with each region represented by a specific color. We circled the right hemisphere on the electronic photos obtained by the scanner as the research object and sequentially obtained the segmentation analysis map of the right hemisphere at different grades.

### 4.10. Statistical Analysis

Statistical analyses were performed using the SPSS Statistics software version 19.0, and the data were expressed as mean ± standard deviation. One-way ANOVA was used to analyze differences between groups and was performed prior to the variance homogeneity test. The least significant difference statistical method was used when the variance homogeneity test was *p* > 0.05, and the Tamhane’s T2 statistical method was used when the variance homogeneity test was *p* < 0.05. Statistical significance was considered when *p* < 0.05.

## 5. Conclusions

This study found that thymoquinone had a preventive and protective function in a model of cerebral ischemia reperfusion injury caused by tMCAO. Thymoquinone alleviated cerebral ischemia–reperfusion injury by reducing the neurological deficit scores, percentage of cerebral infarction, and cerebral edema damage, and increasing the number of active neurons in the ischemic penumbra. Mass spectrometry imaging showed that the ischemic areas were primarily located in the cortex and striatum in the affected side of the brain and that thymoquinone was able to improve the content of various endogenous small molecules after cerebral ischemia–reperfusion injury. Thymoquinone’s protective effect was also shown through its promotion of aerobic oxidation and energy metabolism and regulation of the content of excitatory amino acids, phospholipids, antioxidants, and metal ions.

## Supplementary Materials

The following are available online at https://www.mdpi.com/2218-1989/10/1/27/s1, Figure S1: Negative ion mode mass spectrum of aerobic oxidation-related molecules and amino acids using a 1,5-DAN hydrochloride matrix, Figure S2: Negative ion mode mass spectrum of energy metabolism-related molecules using a 1,5-DAN hydrochloride matrix, Figure S3: Negative ion mode mass spectrum of phospholipid molecules using a 1,5-DAN hydrochloride matrix, Figure S4: Negative ion mode mass spectrum of other metabolic molecules using a 1,5-DAN hydrochloride matrix.
