# Supplementary material for: Effects of Thymoquinone on Small-Molecule Metabolites in a Rat Model of Cerebral Ischemia Reperfusion Injury Assessed using MALDI-MSI
Source: Metabolites. 2020 Jan 7;10(1):27. doi: 10.3390/metabo10010027 (PMC7023359; doi:10.3390/metabo10010027)
Supplement: Supplementary file 1 [file metabolites-10-00027-s001.pdf]

Effects of Thymoquinone on Small-Molecule Metabolites in a Rat Model of Cerebral Ischemia Reperfusion Injury Assessed using MALDI-MSI

Fang Tian [1,2], Runzhe Liu [1,2], Chaoxin Fan [1,2], Yi Sun [1,2], Xi Huang [3,4], Zongxiu Nie [3,4], Xin Zhao [1,2], and Xiaoping Pu [1,2*]

1 National Key Research Laboratory of Natural and Biomimetic Drugs, Peking University, Beijing 100191, China
2 Department of Molecular and Cellular Pharmacology, School of Pharmaceutical Sciences, Peking University, Beijing 100191, China
3 Key Laboratory of Analytical Chemistry for Living Biosystems, Institute of Chemistry Chinese Academy of Sciences, Beijing 100190, China
4 Beijing National Laboratory for Molecular Sciences, Beijing 100190, China
* Correspondence: pxp123@bjmu.edu.cn; Tel: +86-10-82802431

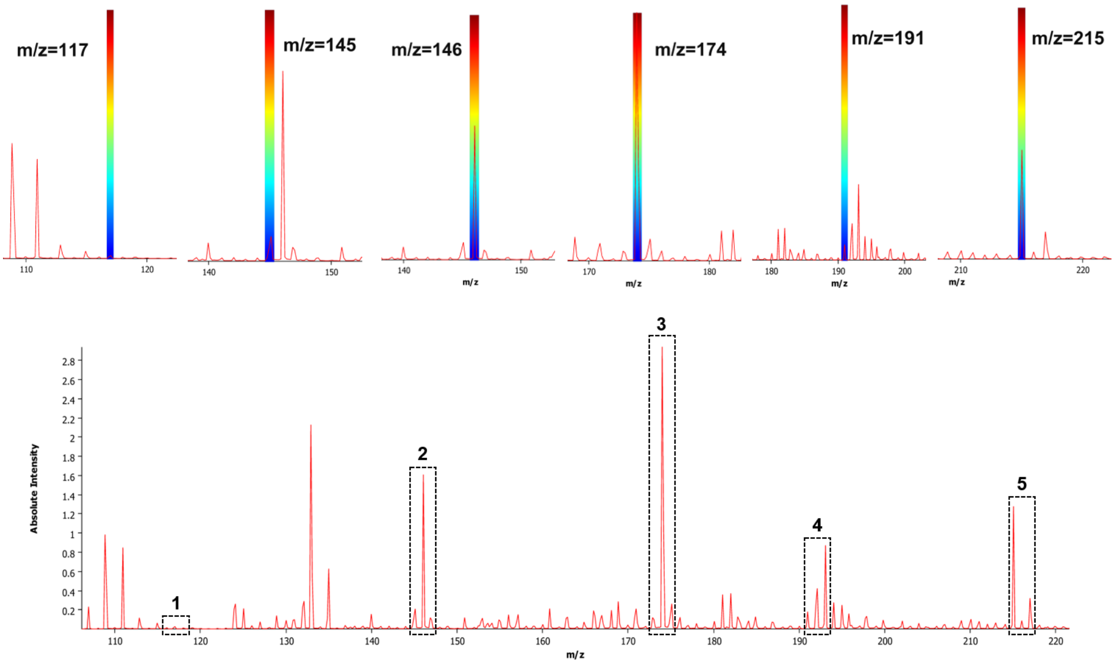

Figure S1. Negative ion mode mass spectrum of aerobic oxidation-related molecules and amino acids using a 1, 5-DAN hydrochloride matrix. Whole mass spectrometry ion peak map with m/z ranging from 110 to 220. Black rectangular areas in the whole mass spectrometry were amplified and displayed. 1: Succinate m/z= 117, 2: Glutamine m/z= 145 and Glutamate m/z= 146, 3: N-acetyl-L-aspartate m/z= 174, 4: Citric acid m/z= 191, 5: Glucose m/z= 215.

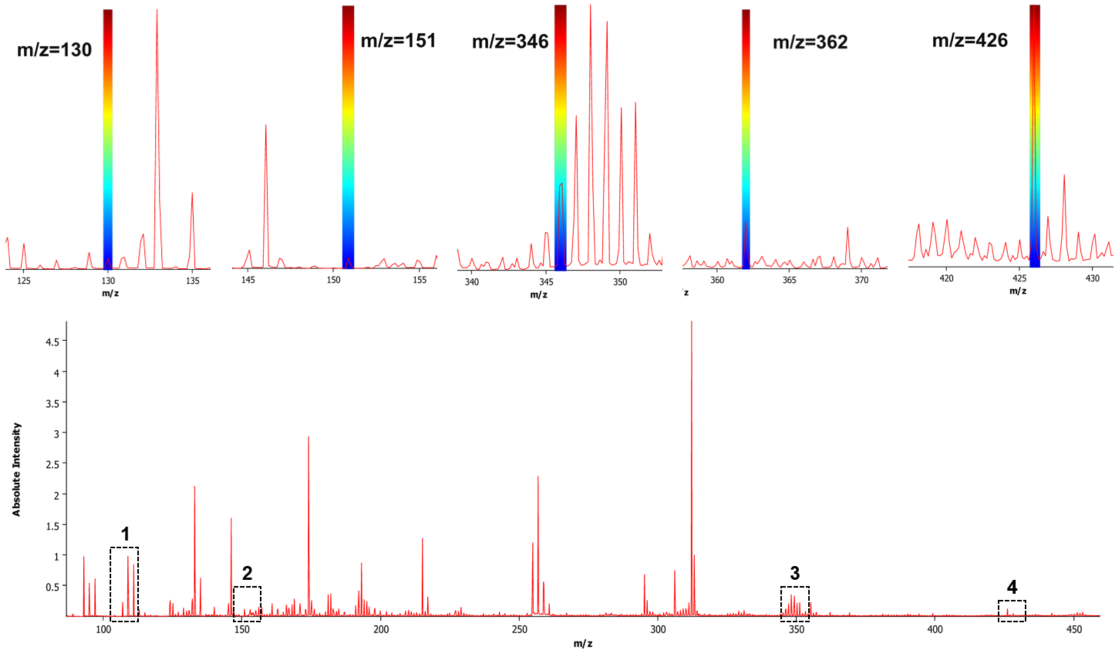

Figure S2. Negative ion mode mass spectrum of energy metabolism-related molecules using a 1, 5-DAN hydrochloride matrix. Whole mass spectrometry ion peak map with m/z ranging from 100 to 450. Black rectangular areas in the whole mass spectrometry were amplifed and displayed. 1: Creatine m/z= 130, 2: Xanthine m/z= 151, 3: AMP m/z= 346 and GMP m/z= 362, 4: ADP m/z= 426.

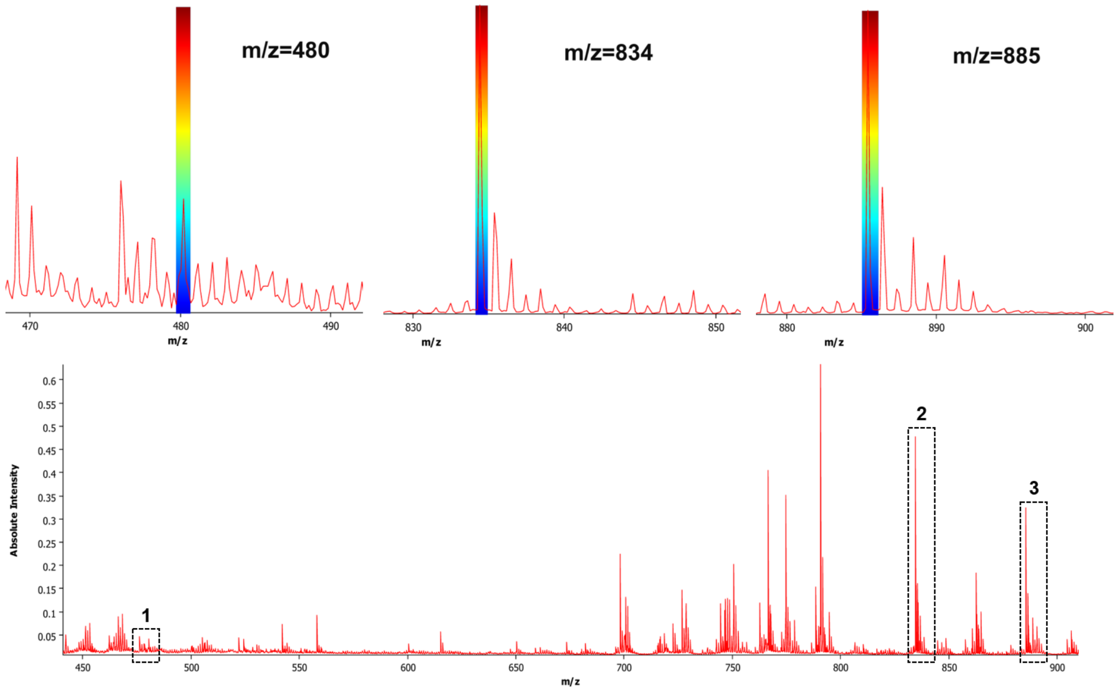

Figure S3. Negative ion mode mass spectrum of phospholipid molecules using a 1, 5-DAN hydrochloride matrix. Whole mass spectrometry ion peak map with m/z ranging from 450 to 900. Black rectangular areas in the whole mass spectrometry were amplified and displayed. 1: PE (18:0) m/z= 480, 2: PS (18:0/22:6) m/z= 834, 3: PI (18:0/20:4) m/z= 885.

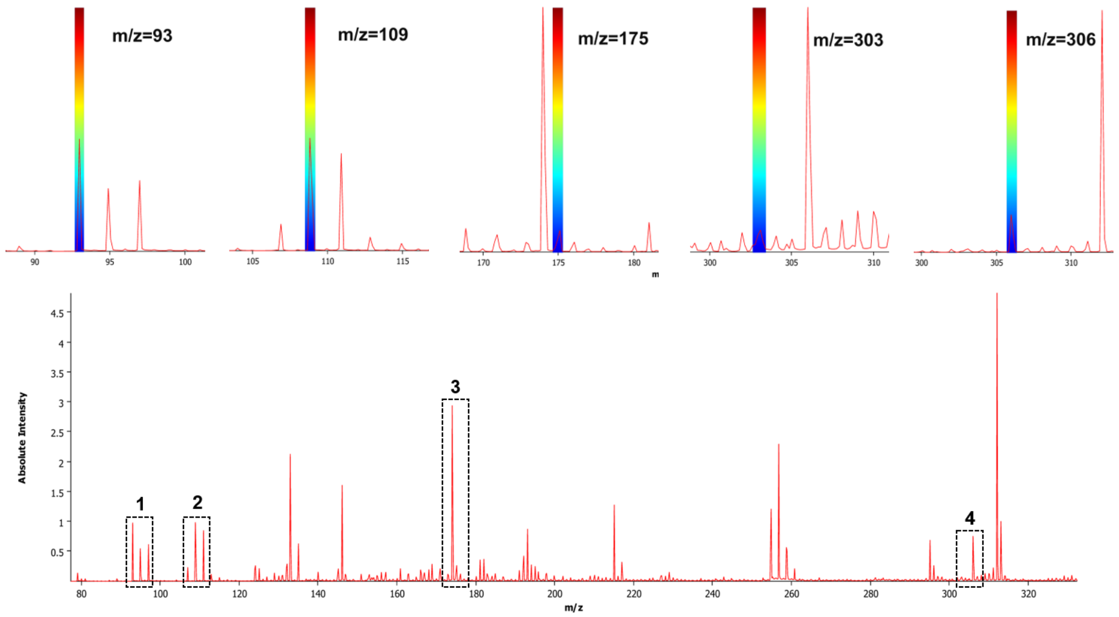

Figure S4. Negative ion mode mass spectrum of other metabolic molecules using a 1, 5-DAN hydrochloride matrix. Whole mass spectrometry ion peak map with m/z ranging from 80 to 320. Black rectangular areas in the whole mass spectrometry were amplified and displayed. 1: Na^+ m/z= 93, 2: K^+ m/z= 109, 3: Ascorbic acid m/z= 175, 4: Arachidonic acid m/z= 303 and Glutathione m/z= 306.
